# Infection control: Knowledge and compliance among Saudi undergraduate dental students

**DOI:** 10.3205/dgkh000253

**Published:** 2015-07-01

**Authors:** Sadeq Ali Al-Maweri, Bassel Tarakji, Bassam Shugaa-Addin, Hashem M. Al-Shamiri, Nader Ahmed Alaizari, Ousamah AlMasri

**Affiliations:** 1 Department of Oral and Maxillofacial Sciences, Al-Farabi Colleges of Dentistry and Nursing, Riyadh, Saudi Arabia; 2 Department of Oral Medicine and Diagnosis, Sana’a University, Sana’a, Yemen; 3 Al-Farabi Colleges of Dentistry and Nursing, Riyadh, Saudi Arabia

**Keywords:** knowledge, compliance, infection control, dental students

## Abstract

**Objective:** This study aimed to assess the level of knowledge, attitudes, and practices regarding infection control procedures among undergraduate dental students.

**Methods:** This was a questionnaire-based cross-sectional survey. A self-administered questionnaire consisting of questions on students’ vaccination status as well as knowledge and attitudes regarding infection control was sent to 600 undergraduate dental students in the fourth, fifth, and sixth year of the Al-Farabi College for Dentistry and Nursing, Riyadh, Saudi Arabia. The collected data were analyzed using SPSS software. The significance level was set at *P*<0.05.

**Results:** The response rate was 85% (512 out of 600). While the vast majority of students (90%) had been vaccinated against hepatitis, only 37.4% have been assessed for anti-HBs. A total of 98.8% and 90.8% reported always wearing gloves and masks, respectively, during dental procedures. The use of protective eyewear was reported by only 29.2%. A significantly higher proportion of sixth-year students showed a positive attitude toward the treatment of patients with infectious diseases than other students of lower academic years. Approximately one-third of students reported having one or more occupational injuries while treating their patients.

**Conclusion:** Although the students had good knowledge and attitudes regarding infection control, the compliance and practice levels regarding the same were low. Such findings highlight the necessity of continued infection-control education of Saudi dental students.

## Zusammenfassung

**Zielsetzung:** In der Studie sollten die Kenntnisse, die Einstellung und die praktische Umsetzung infektionspräventiver Maßnahmen bei Studenten der Zahnmedizin analysiert werden.

**Methode:** In einer Querschnittsstudie wurde ein selbst zu beantwortender Fragebogen zur Erfassung des Impfstatus, der Kenntnisse und der Einstellung zur Infektionsprävention an 600 Studenten im 4., 5. und 6. Ausbildungsjahr des Al-Farabi College für Dentistry und Nursing, Riyadh, Saudi Arabia, verschickt. Die Daten wurden mittels SPSS analysiert. Das Signifikanzniveau wurde mit p<0,05 festgelegt.

**Ergebnisse:** Die Responserate betrug 85% (512 von 600). Während die Mehrzahl der Studenten (90%) gegen Hepatitis B geimpft war, war nur bei 37,4% anti-HBs bestimmt worden. 98,8% bzw. 90,8% trugen stets Schutzhandschuhe bzw. Mund-Nasen-Schutz bei der zahnärztlichen Behandlung, während das Tragen einer Schutzbrille nur in 29,2% angegeben wurde. Ein signifikant höherer Anteil der Studenten zeigte im 6. Ausbildungsjahr eine positive Einstellung zur Behandlung von Patienten mit Infektionskrankheiten im Vergleich zum 4. und 5. Ausbildungsjahr. Etwa ein Drittel der Studenten gab an, dass sich ein oder mehrere arbeitsbedingte Verletzungen bei der Patientenbehandlung ereignet hatten.

**Schlussfolgerung:** Obwohl die Studenten ein solides Wissen und eine gute Einstellung zur Infektionsprävention hatten, war die Compliance zur praktischen Umsetzung gering. Die Ergebnisse unterstreichen die Notwendigkeit der kontinuierlichen Erziehung Saudischer Zahnmedizinstudenten zur Infektionsprävention.

## Introduction

Dental health personnel (DHP) including dental students are at high risk of exposure to cross-infection with blood-borne pathogens, such as hepatitis B virus (HBV) and hepatitis C virus (HCV), as well as with other viruses and bacteria that colonize the oral cavity and the upper respiratory tract, such as influenza virus, *Mycobacterium tuberculosis*, and streptococci [1], [2], [3]. This risk is enhanced by occupational percutaneous injuries and eye exposure caused by dental instruments during dental treatment. Such infections can be prevented by using safety precautions and implementing infection control guidelines coupled with vaccination and proper post-exposure management. 

Cross-infection can be defined as the transmission of infectious agents between patients and staff within a clinical environment [4]. Direct involvement in patient treatment as part of their clinical training puts dental students at risk of exposure to pathogens. As the majority of carriers of infectious diseases cannot be identified clinically, implementation of standard universal precautions in dental schools is the most effective way to control cross-infection [5], [6].

Dental institutes are responsible for providing appropriate infection control measures, proper training of dental students to protect patients, and the establishment of safer working conditions [7]. In 2003, the US Center for Disease Control and Prevention (CDC) updated its guidelines on infection control in dental settings [6]. These guideline includes standard precautions which aim to ensure a safe working environment and prevent the potential transmission of occupational and nosocomial infections among DHP and their patients. Unfortunately, despite the considerable emphasis placed on the importance of adherence to these protocols, studies have shown that few dentists actually adhere to the standardized infection control procedures in their daily practice [4], [8], [9], [10], [11]. Moreover, several studies worldwide have investigated undergraduate dental students’ knowledge and attitudes regarding infection control, finding very poor compliance with infection control guidelines and indicating the need to improve the knowledge and attitudes regarding infection control [3], [7], [12], [13], [14], [15]. To date, no published data exists regarding infection control practices among dental students in Saudi Arabia. In light of this paucity of data, the aim of this study was to assess the knowledge, attitudes, and practices regarding infection control among dental students at Al-Farabi colleges of Dentistry and Nursing, Riyadh, Saudi.

## Methods

This study, conducted in December 2014, consisted of a cross-sectional survey of dental students at the School of Dentistry, Al-Farabi Colleges, Riyadh, Saudi Arabia. All clinical students (4^th^–6^th^ year) enrolled during the 2014–2015 academic year were eligible to participate. The study was approved by the Al-Farabi College Institutional Ethical Review Board. We used a self-administered questionnaire, adapted from a pretested questionnaire that has been applied in similar studies [3], [7]. A pilot study was conducted on a random sample of students (n=40) to ensure that the questions were understandable; the questionnaire was modified according to the feedback obtained. Participation in the study was completely voluntary. Participants were informed that they could withdraw at any time, and that their responses would be anonymous and treated confidentially. 

The questionnaire consisted of 13 open- and close-ended questions related to hepatitis B vaccination and serology, the use of personal protective equipment, infection control practices and awareness, percutaneous and eye exposure, and attitudes toward the dental treatment of infected patients. Questionnaires were distributed to students during the clinical sessions.

Data management and statistical analysis were performed using the statistical software SPSS version 20.0. Frequencies and percentages were obtained for categorical data, and the Chi-square test was used to determine the association between variables. A *p*-value <0.05 was considered significant.

## Results

Out of the invited 600 students for the study, 512 returned the completed questionnaires with an overall response rate of 85%. The mean age of the participants was 23±3.28 years (range: 20–40 years). More than half (54.1%) of the participants were female. The sample comprised an almost equal distribution of 4^th^-, 5^th^- and 6^th^-year students (Table 1 (Tab. 1)). 

Vaccination was completed by an almost 90% of the students with a significant difference between males and females (82.6% vs. 95%). There was a positive correlation between vaccination status and academic year (p<0.05). Out of the vaccinated students, only 67.7% completed the recommended 3 doses of vaccination, with significant differences according to academic year (p<0.001). No correlation was found between number of doses and gender (p>0.05). Only around one-third of the students (36.7%) were tested for post-HBV immunization, with no significant differences according to gender or academic year (p>0.05; Table 2 (Tab. 2)).

Table 3 (Tab. 3) shows students’ self-reported use of protective barrier techniques. The vast majority reported always wearing gloves (98.5%), gowns (91.6) and masks (90.8%). On the other hand, only 32.5% of fourth-year, 31% of fifth-year, and 23.8% of sixth-year students always used protective eyewear, while the majority used it occasionally. No correlation was found between use of protective barrier techniques and gender of students (p>0.05).

While the vast majority of students (99.6%) reported changing gloves between patients, only 73.2% reported disinfecting their hands after each gloves change, with no significant differences between fourth-, fifth, and sixth-year students (p>0.05). In this study, 97% of the students thought that dental schools bear the responsibility for implementing infection control recommendations, and around 96.1% were planning on following the same infection control procedures in their clinics/practices after graduation (Table 4 (Tab. 4)). 

**Figure 1:**
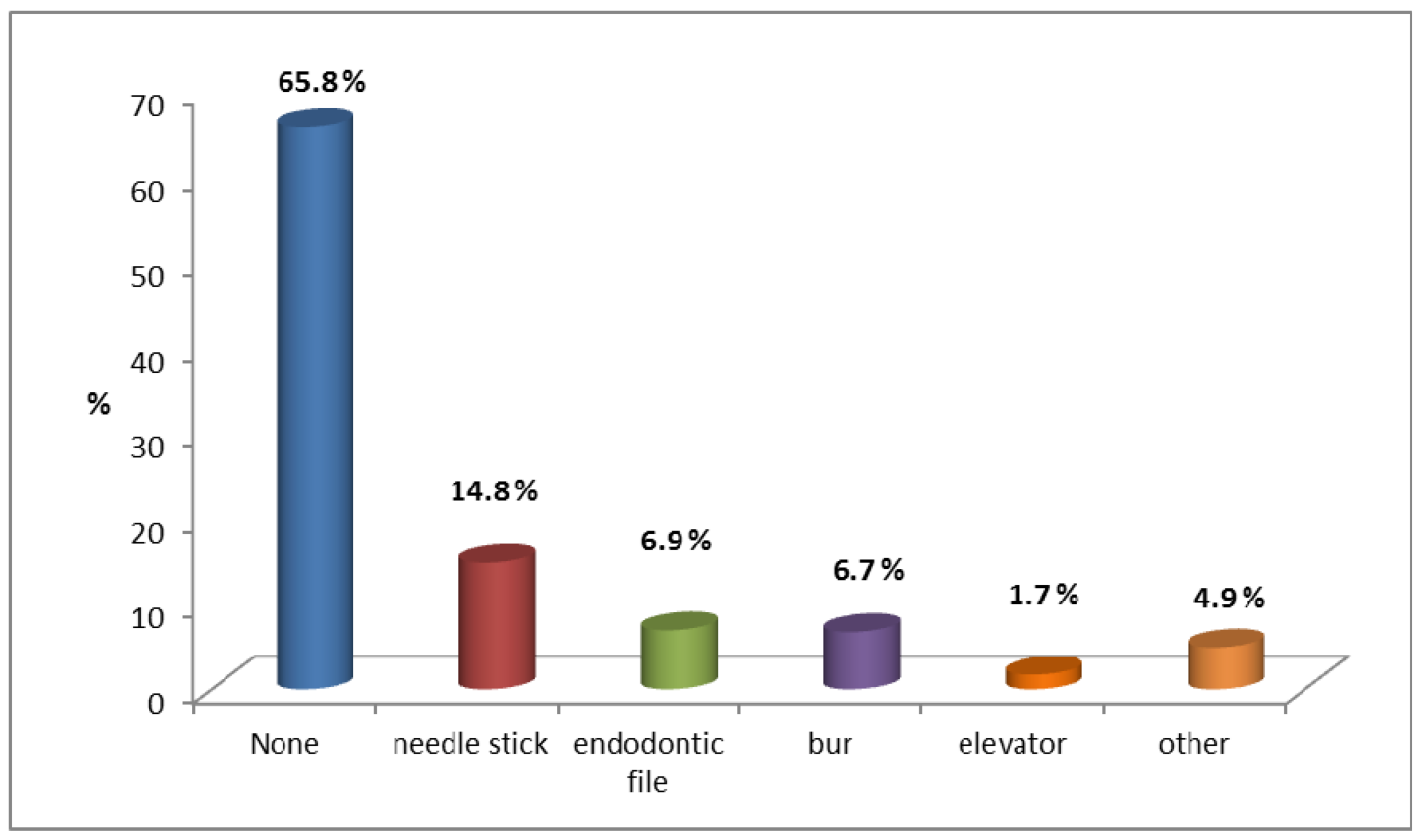
Proportion of exposures caused by different instruments

Non-sterile occupational percutaneous injuries and splashes to the eye were reported by around one-third of students (34.2%), with significant differences between students in different years of study (p<0.001). Most of the reported injuries were caused by anesthesia needles and endodontic files (Figure 1 (Fig. 1)).

A significantly higher proportion of final-year students (62.3%) showed a positive attitude towards the treatment of patients with infectious diseases compared to only 45.7% and 58.1% of fourth- and fifth- year students, respectively (p<0.01). Male students showed a better attitude towards treatment of patients with infectious diseases as compared to females (61.1% vs. 49.3; p<0.01). Likewise, a considerably higher number of sixth-year students reported having treated one or more patients with infectious diseases as compared to other students of lower academic years. Most of the treated patients with infectious diseases had hepatitis B.

## Discussion

Dental health professionals are at high risk of infection by blood-borne pathogens, as they are continually exposed to blood and saliva mixed with blood, and may even suffer needle punctures [11], [16]. The key to reducing or preventing the transmission of a variety of microorganisms to dental workers lies in strict adherence to infection control practices. This study reports the overall knowledge, attitudes, and practices regarding infection control among dental students at Al-Farabi Colleges for Dentistry and Nursing, Riyadh, Saudi Arabia. To the best of our knowledge, this is the first study investigating the topic among dental students in Saudi Arabia. Overall, dental students in the present study had good knowledge and positive attitudes regarding infection control, but showed poor compliance with the recommended infection control guidelines. These results were also found in other studies [3], [7], [17]. We also noticed a higher level of knowledge of and compliance with infection control practices among students of higher academic levels.

Proper hepatitis B vaccination is the best procedure to prevent contagious transmission during dental treatments [7]. The prevalence of hepatitis B vaccination among dental health workers varies from 38% to 100% [3], [7], [13], [14], [15], [16], [17]. In the present study, the majority (90%) reported having been vaccinated against hepatitis B. However, only two-thirds of our students completed the 3 recommended doses. A similar finding was reported by Rahman et al. [65%] [3]. However, this rate is much lower than that reported in other studies by De Souza et al. [7], Alavian et al. [17], and Kramer et al. [18], in which more than 80% of students received the required 3 doses of HBV vaccination. Notably, the present authors observed that female students completed the immunization more frequently than did their male counterpart, which is in agreement with the literature [7].

The efficacy of HBV vaccination can be assessed by post-immunization HB titer level. Unfortunately, only 34.5% of students who were immunized reported post-HBV immunization serology, a finding similar to the results found by other studies. In a survey of dental students, De Souza et al. [7] found that 90.8% were vaccinated. However, only 27.5% reported post-HBV immunization serology. In a survey of students in professional health care fields, McCarthy and Britton [13] found that 100.0% of dental, 98.7% of medical, and 95.3% of nursing students were vaccinated. However, a significant proportion of students failed to confirm the adequacy of their post-immunization anti-HBs titer. Although the hepatitis B vaccination rate among students of this sample was high, universal vaccination and serological testing should be encouraged to reduce the risk of acquiring hepatitis B following an occupational exposure.

The practice of standard precautions including the use of barrier techniques has been shown to be the best prevention strategy against occupational transmission of infectious diseases in health care settings. In the present study, there was high compliance with glove and mask use, similar to previous studies conducted in Canada, Germany, the UK, Iran and UAE [3], [9], [13], [16], [17], [18], [19]. Nonetheless, compliance with protective eyewear was very low; only one-third reported using protective eyewear at all times. This unsatisfactory result, however, is not peculiar to Saudi Arabia, as many other studies in the UK, UAE, and Nigeria have also shown that a majority of dental students did not use eye protection most of the time [3], [14], [19]. Similarly, in a German multicenter study only 64% of dentists wore protective goggles or other eye protection during treatment [18]. The poor utilization of eyewear and face masks may indicate a low level of awareness among students and dentists about the probability of disease transmission via aerosols and blood splashes. Hence, dental students should be encouraged to wear masks and protective eyewear to minimize the chance of transmitting airborne infections.

The transmission of pathogens from the hands of dental personnel to patients is of major concern for infection control. Hand hygiene is considered the single most effective method for the prevention and control of healthcare-associated infections [16]. Compliance with hand hygiene procedures is essential, as the hands of healthcare workers may serve as reservoirs for many pathogens [16], [20], [21]. In the present study, however, compliance with hand hygiene procedures was unsatisfactory, as only 70% of our students reported washing their hands after each glove change. This finding is consistent with earlier studies conducted by Rahman et al. [3], De Amorim-Finzi et al. [22] and Kramer et al. [18]. In the latter study, hand disinfection was done before each treatment only in 20–50%. Moreover, a recent study among dental healthcare personnel in a German university dental clinic found a very low compliance with hand hygiene [16]. This low compliance with regular hand disinfection necessitates stricter measures to remind the students of the importance of hand disinfection. For example, disinfectant dispensers can be placed near each basin in dental clinics. At our college, a newly appointed infection control officer – not one of the scheduled clinical instructors – makes rounds in every clinical session to ascertain implementation of infection control in student clinics.

Approximately one-third of the students mentioned that they had non-sterile occupational injuries. This figure is very close to the 30.5% prevalence rate reported in a similar study conducted among dental students in Brazil [7]. However, this rate is much lower than that reported among dental students in Canada, in which over 80% of students reported certain types of injuries [13]. Such alarming findings demonstrate that dental students are at a high risk of developing serious infections with blood-borne pathogens, including HIV infection. For instance, it has been found that the estimated risk of acquiring infection with hepatitis B from a percutaneous exposure ranges from 5% to 45% [19]. We noted that the prevalence of accidental injuries was highest among final-year students (sixth year) and lowest among fourth-year students, probably due to the fact that sixth-year students had longer clinical exposure than did the 4^th^-year students. This result supports previous studies [3]. Also, consistent with the findings of other authors, the anesthetic needle was the major source of accidental injuries in our survey [3], [7], [13], [23]. Sharp injuries are more likely to occur in the dental environment than in other healthcare settings [23], usually due to the small operating field, frequent patient movements, and the variety of sharp dental instruments. Such injuries may pose the risk of transmission of blood-borne pathogens, especially hepatitis B, C, or HIV [7], [9], [23].

In the present study, fifth-year students showed a more positive attitude towards treating patients with infectious diseases than other students of lower academic years, which is in accordance with previous studies [3], [17]. This relative improvement in attitude toward patients with infectious diseases may suggest that theoretical and practical training in HBV and HIV protection can improve students’ attitudes about treating these patients. Also, in agreement with a previous study conducted by Alavian et al. [17], we found a significant correlation between gender and the willingness to treat patients with infectious diseases (female students were more willing to do so).

Comparing the present findings with the those of our similar previous study conducted among dental students in Yemen [24], there were both major differences and some similarities. The vaccination rate in the present study was much higher than the rate reported among Yemeni dental students (90% vs. 71.7%). This could be attributed to the fact that vaccination against HBV is considered a mandatory requirement by the dental and medical schools in Saudi Arabia but not in Yemen, which in turn explains the low vaccination rate found in the latter study. This emphasizes the importance of making all vaccinations, especially hepatitis B, mandatory for students prior to granting admission to any dental institution. The reported use of protective barriers was higher in the present study than in the previous study in Yemen: facemask 90.8% vs. 53.8%,and eyewear 29.2% vs 14%. Moreover, the compliance with hand hygiene procedures was also much higher in the present study (70%) compared with the findings for Yemeni students (43%), although the results of both studies were unsatisfactory. Unsurprisingly, percutaneous injuries were much lower in the present study (33.5%) as compared to that reported in the previous study in Yemen (62.3%), a finding which can be ascribed to the students’ lack of experience with infection control procedures and also to the fact that students in Yemen usually work on patients without assistance, while Saudi students must always work on patients with assistance. The students in both studies showed a similar attitude towards treatment of patients with infectious diseases.

Despite the large sample size and high response rate of the present survey, there are several potential limitations that should be taken into consideration. One limitation is the fact that the responses were subjective (i.e. based on students’ self-report) rather than being provided under supervision in a clinical environment, and therefore the results may not necessarily fully reflect students’ real knowledge and daily professional practice. Additionally, this number of questions cannot show the real knowledge and practice of the respondents. Nevertheless, the number of questions was kept to a minimum to improve the response rate, which appeared to work well. Despite these limitations, however, this study provides some important information about Saudi dental students’ knowledge, opinions, and practices regarding infection control. Such information should help identify areas that need reinforcement or greater emphasis in the dental curriculum.

## Conclusion

In conclusion, dental students in the present study showed a good level of knowledge and positive attitudes about infection control. However, the knowledge acquired must be transferred into daily practice. With all infection control protocols already implemented in dental schools, improving compliance with infection control recommendations remains a challenge. Compliance can be improved by refreshing and upgrading students’ knowledge through seminars or lectures on universal infection control measures each academic year, and by keeping all vaccinations mandatory, especially hepatitis B, for students prior to being admitted to any dental institution.

## Notes

### Competing interests

The authors declare that they have no competing interests.

## Figures and Tables

**Table 1:**
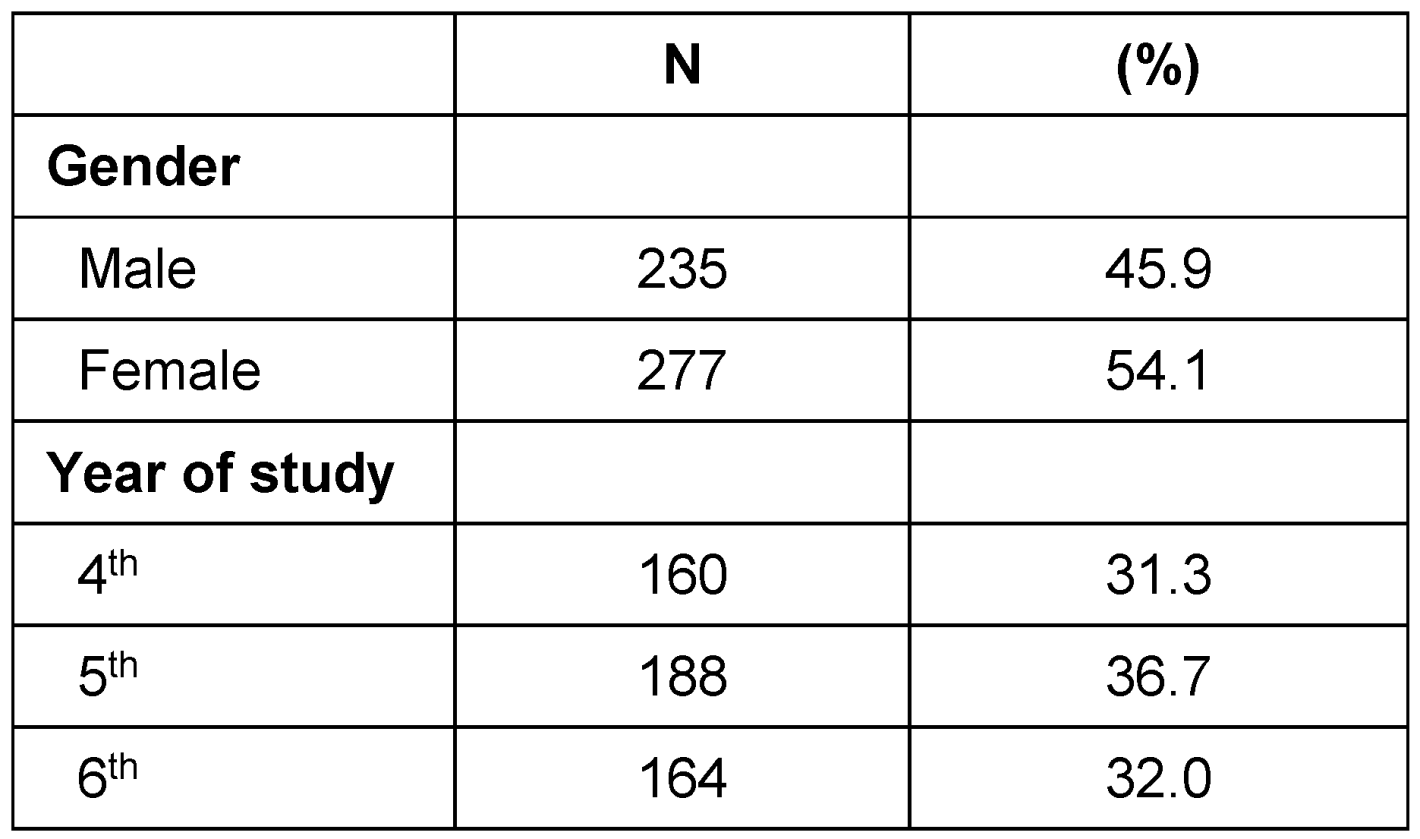
Distribution of the students according to gender and the academic level

**Table 2:**
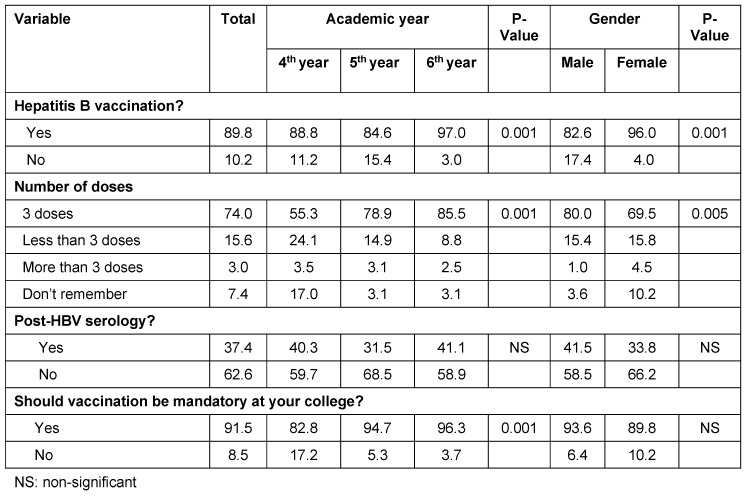
Proportion of vaccination, number of doses and post-HBV serology among the subjects

**Table 3:**
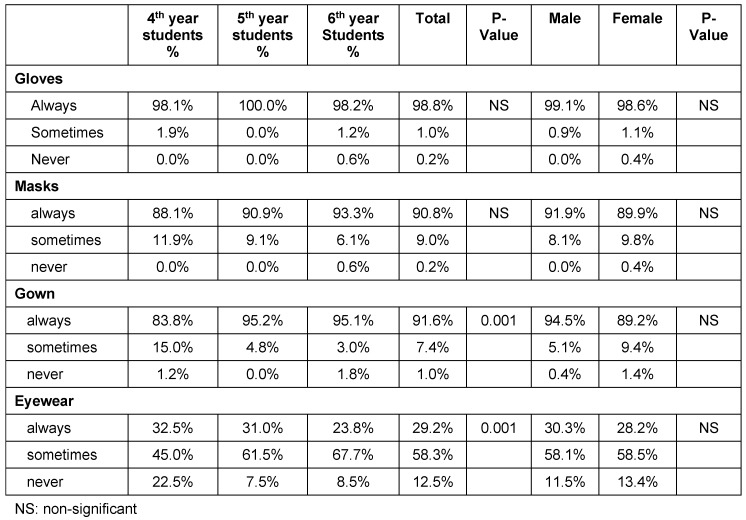
Use of protective barrier techniques among subjects by gender and year of study

**Table 4:**
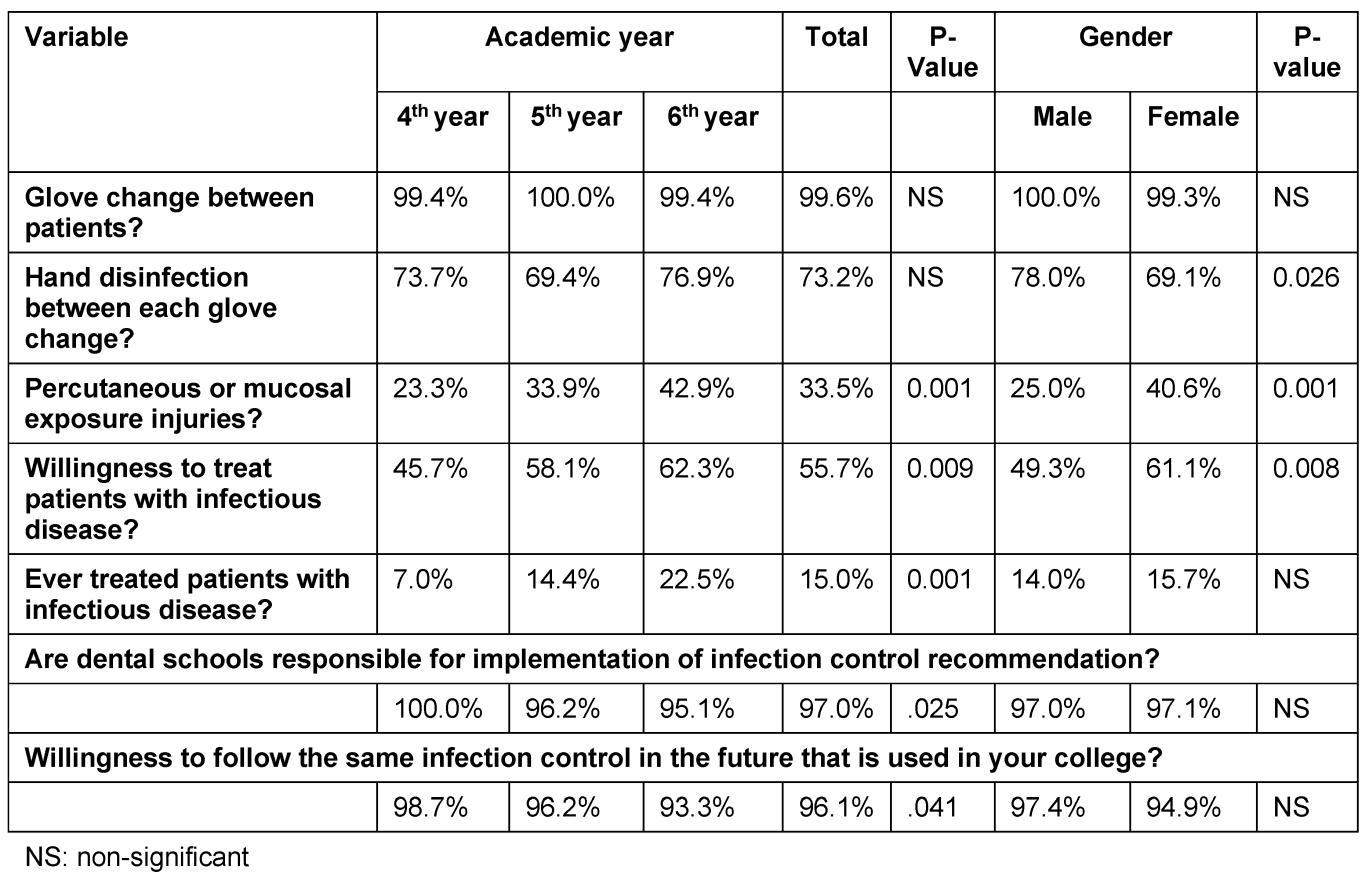
Practices and attitudes of students by gender and academic year
